# Cancer and Post‐Therapy Cardiotoxicity Risk in Adolescents, Young Adults, and Adults With Down Syndrome

**DOI:** 10.1002/cph4.70037

**Published:** 2025-09-05

**Authors:** Michelle A. Buckman, Anastasiia Vasileva, Charles R. Jedlicka, Hardik Kalra, Mikhail Vasilyev, David S. Dickens, Michael H. Tomasson, Melissa L. Bates

**Affiliations:** ^1^ Department of Internal Medicine University of Iowa Iowa City IA USA; ^2^ Department of Health and Human Physiology University of Iowa Iowa City IA USA; ^3^ Department of Pediatrics University of Iowa Iowa City IA USA

**Keywords:** anthracyclines, cardio‐oncology, leukemia, malignancy, multiple myeloma, myelodysplastic syndrome

## Abstract

The median life expectancy of people with Down syndrome has increased substantially over the past several decades, from 4 years in 1970 to 53 years in 2010. Despite the recent improvement in survival, there is little data about the prevalence of age‐related diseases, including age‐related malignancies, and the impact of standard cancer treatments on cardiovascular health. We retrospectively reviewed medical records for age‐ and sex‐matched patients ≥ 15 years old with and without Down syndrome using the TriNetX platform to identify the prevalence of malignancies and explore cardiovascular outcomes after treatment with anthracyclines. We further stratified the populations into adolescent and young adult (AYA, ages 15–39 years old) and adult (≥ 40 years old) cohorts, given that treatment recommendations can be different. Down syndrome patients in the AYA cohort were more likely to be diagnosed with acute myeloid leukemia (OR 8.9, CI 4.99–15.89, *p* < 0.001) and lymphoid leukemia (OR 7.33, CI 4.82–11.15, *p* < 0.001) The adult cohort with Down syndrome was more likely to be diagnosed with myelodysplastic syndromes (OR 12.25, CI 6.41–23.42, *p* < 0.001), multiple myeloma (OR 1.66, CI 1.06–2.6, *p* = 0.026), and testicular cancer (OR 2.73, CI 1.32–5.65, *p* = 0.005). Overall, Down syndrome patients (≥ 15 years old) treated with anthracyclines were more likely to be diagnosed with heart failure (OR 2.14, CI 1.07–4.27, *p* = 0.042). Our study demonstrates adolescents and adults with down syndrome have a higher predisposition to several malignancies and an increased risk of cardiovascular disease after anthracycline treatment and may require specific screening guidelines to address their unique health risks.

## Graphical abstract

Adults with down syndrome are more likely to develop hematological malignancies and testicular cancer. Additionally, they are more susceptible to cardiovascular diseases after anthracycline treatment. Created with BioRender.com.

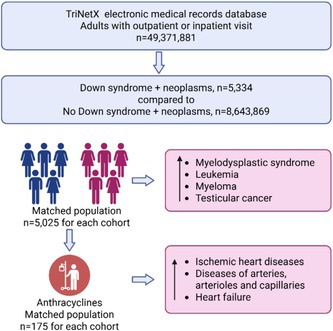

## 1 Introduction

Since 1950, there has been a four‐fold increase in the population prevalence of down syndrome (DS) in the United States, with a current frequency of 1 in 1499 live births (De Graaf et al. 2017). Since 1970, the median age at death has increased from 4 to 53 years, such that the DS population is both larger and older than in previous decades (Presson et al. 2013). This increase in life expectancy can be attributed to reduced institutionalization, advancement in surgical treatments of congenital heart disease, and better‐established healthcare guidelines, including routine physical and laboratory examinations for children with DS (De Graaf et al. 2017; Antonarakis et al. 2020; Bull 2011) Given the increase in the median life expectancy in DS, it is necessary to understand their susceptibility to age‐related comorbidities and tailor screening and treatment guidelines to meet the needs of this understudied population.

The imbalance in gene dosage from the triplication of human chromosome 21 is associated with accelerated aging (Zigman 2013). This is partly due to the overexpression of oxidative stress‐related genes on human chromosome 21, including *SOD1*, *ETS2*, *S100*, and *NIRP1* (Muchová et al. 2001; Helguera et al. 2005; Esposito et al. 2008; Izzo et al. 2014). Age‐related cancer risk is under‐explored. Most studies of cancer prevalence and DS focus only on children or do not stratify the population by age for analysis (Hasle et al. 2000; Patja et al. 2006; Hill et al. 2003). Krieg et al. found no increased risk of cancer in adults with DS (Krieg et al. 2024); but this study focused only on cancers observed in their study population after five years and, therefore, the sample size and the limited 5‐year follow‐up period may have limited the ability to detect population‐level differences, particularly in rarer malignancies. Hasle et al. found a lower‐than‐expected incidence of breast cancer in individuals with DS, but the small cohort (113 cancer cases overall) limits the ability to evaluate risk except for the most common malignancies (Hasle et al. 2016). The current data are insufficient to develop comprehensive, age‐specific cancer screening guidelines for the DS population (Rethore et al. 2020; Tsou et al. 2020).

Anthracyclines are a class of anti‐neoplastic drugs that inhibit DNA synthesis in rapidly dividing cells and are commonly used to treat blood and solid tumor cancers (Henriksen 2018). Chronic cardiotoxicity is a serious side effect of anthracycline treatment, and survivors of childhood leukemia with DS are at an increased risk of heart failure (Krischer et al. 1997; Hefti and Blanco 2016). However, the effect of anthracyclines on the cardiovascular system of individuals with DS > 15 years old is unknown. In the general population, anthracycline‐induced cardiovascular toxicity clinically manifests as left ventricular dysfunction, congestive heart failure, cardiomyopathy, cardiac arrhythmias, and/or alterations in cardiac structure (Zamorano et al. 2016). We previously found that adults with DS are at a higher risk of hypertension, hypotension, cerebrovascular disease, and ischemic heart disease compared to age‐ and sex‐matched controls, and that risk factors used to predict risk in the control population are not necessarily predictive of cardiovascular disease risk in DS (Bates et al. 2023). Given the overall increased cardiovascular disease risk in DS and the lack of predictive value of typical risk factors, it is critical to independently identify whether they are at an increased risk of chronic cardiotoxicity with anthracycline therapy. Furthermore, adolescent and young adult (AYA) patients are often treated with pediatric‐inspired regimens that can include increased exposure to anthracyclines (Janardan and Miller 2023), which may alter cardiovascular risk. This highlights the importance of studying the nonpediatric DS population as two cohorts whenever possible—AYA (age 15–39 years) and adult (≥ 40 years).

This study's purpose was to comprehensively investigate the prevalence of solid and hematological malignancies in AYA and adult patients with DS by retrospectively analyzing a large medical record database. We further tested the hypothesis that adults with DS who have a history of treatment with anthracyclines will have a higher prevalence of posttreatment cardiovascular disease compared to adults without DS.

## 2 Methods

### 2.1 Study Design and Patient Population

A retrospective review of medical records was conducted in January 2025 using the research network of the TriNetX platform, an electronic medical records database consisting of de‐identified patient information that can be accessed in aggregate. At the time of the study, the TriNetX platform comprised 117,229,846 adults from 103 healthcare organizations in five countries, mostly in the United States. Data on the TriNetX platform include ICD‐10‐CM (International Classification of Diseases, 10th Revision, Clinical Modification) diagnosis codes, demographics, medications, lab results, and procedure codes. This study was determined to be exempt from review by the Institutional Review Board at the University of Iowa as data are only reported in aggregate, and identifying personal‐level data is not available.

Retrospective medical records were reviewed in aggregate for patients ≥ 15 years old, with the first documented outpatient or inpatient care services as the index event for future follow‐up. These patients were sub‐grouped into two cohorts: patients with and without DS. The ICD10 code for DS identified individuals with DS. Site‐specific cancers and cardiovascular disease types were also determined using ICD‐10 diagnosis codes (Table S1).

### 2.2 Data Analysis

The cohorts were matched based on their age at the index event and female sex using 1:1 propensity score matching with the balance cohorts function of TriNetX. We evaluated the incidence of malignant neoplasms occurring in the primary organ systems of both cohorts, followed by restratification of the cohorts into groups aged 15–39 (AYA) and ≥ 40 (non‐AYA) years old. The age cut‐off for the AYA population was defined according to the National Cancer Institute's definition of individuals diagnosed with cancer between the ages of 15 and 39 years (Page et al. 2025). We then explored the incidence of subtypes of hematologic and solid malignancies recommended for screening and treatment by the American Cancer Society. The TriNetX software automatically eliminated patients who met the index event over 20 years ago. The time window for cancer prevalence started on the day of the first inpatient or outpatient visit after meeting the cohort age criteria, and for cardiovascular disease, the day after the first documented anthracycline treatment, with no designated end date. We further excluded all patients with a neoplasm diagnosis before the index event so that only diagnoses that occurred at or after age 15 were captured. A measure of association analysis, which compares the fraction of patients with the selected neoplastic outcome, was performed using the analysis function of the TriNetX platform. Data are reported as the odds ratio for each neoplasm diagnosis and the 95% confidence intervals. TriNetX automatically reports an outcome as 10 when it involves fewer than 10 patients to protect patient privacy.

Sub‐group analyses were performed to evaluate the incidence of cardiovascular diseases in both cohorts after treatment with anthracyclines. The cohorts were matched based on age at the index event, in this case, the anthracycline treatment, and female sex. The time window for each outcome was set to the day after the index event. All patients diagnosed with an outcome before the index event were excluded. The incidence of cardiovascular diseases in the cohort with DS was compared to the group without DS and is reported as the odds ratio and 95% confidence interval. Given the heightened risk of cardiovascular disease we previously reported (Bates et al. 2023), we performed a within‐cohort analysis to evaluate the incidence of cardiovascular diseases in age‐ and sex‐matched control and DS patients, comparing those with neoplasms and anthracyclines to those without a record of any neoplasm or anthracycline medication. The index event for the cohort with neoplasms was when they had a record of anthracycline treatment, while that of the other group was when they had a record of an outpatient/inpatient visit.

### 2.3 Statistics

Statistical significance was set a priori as *p* < 0.05. All statistical analyses were conducted using the analytics feature in the TriNetX research network. The TriNetX software calculated t‐tests of demographic variables and the odds ratios and 95% confidence intervals for outcomes using the measure of association analyses feature. Figures and all other statistical analyses for significance were generated with the GraphPad Prism 10.0 software package.

## 3 Results

### 3.1 Overall Cohort Characteristics

**FIGURE 1:**
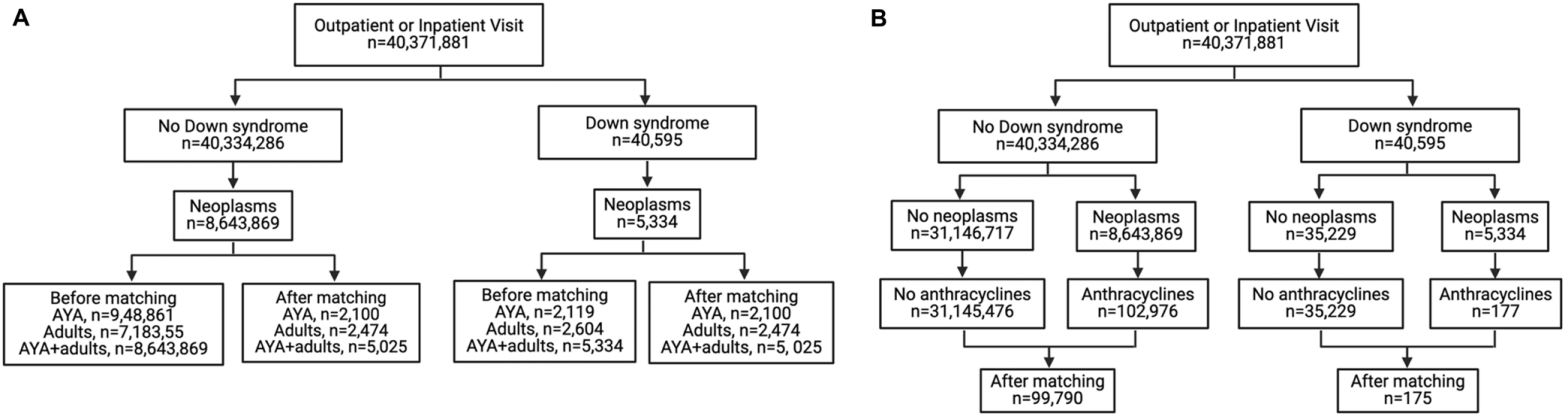
Flow diagram for TriNetX analyses. AYA, adolescent and young adult. Created with BioRender.com.

A total of 40,371,881 patients with outpatient or inpatient visits were identified, and a flowchart of the analysis is outlined in Figure 1. Patient demographics, comorbidities, and lifestyle factors are outlined in Table 1. The age at index (37.1 ± 16.0 years) and female sex (2600, 51.7%) were not different between groups. In the controls, 2182 (43.4%) were male, while the cohort with DS consisted of 2188 (43.5%) male patients. Comorbidities investigated in the controls and the DS cohort include congenital malformations of the circulatory system (73, 1.5% and 930, 18.5%), diabetes (300, 6.0% and 498, 9.9%), pre‐existing hypotension (87, 1.7% and 372, 7.4%), and preexisting hypertension (694, 13.8% and 702, 14.0%). Also, lifestyle factors such as alcohol use disorders (115, 2.3% and 25, 0.5%) and tobacco use (115, 2.3% and 30, 0.6%) were less common in the cohort with DS.

**TABLE 1:** Baseline characteristics of patients with neoplasms before and after matching.

	Before matching			After matching		
	Controls, *n* = 8,643,869	DS, *n* = 5334	*p*	Controls, *n* = 5025	DS, *n* = 5025	*p*
**Age, years**						
Age at index^a^	54.7 ± 16.3	37.1 ± 16.0	**< 0.001**	37.1 ± 16.0	37.1 ± 16.0	1.000
Current age	62.0 ± 16.5	44.6 ± 16.8	**< 0.001**	44.7 ± 16.7	44.6 ± 16.8	0.712
**Sex**						
Female^a^	4,550,445 (55.7%)	2600 (51.7%)	**< 0.001**	2600 (51.7%)	2600 (51.7%)	1.000
Male	3,261,730 (39.9%)	2188 (43.5%)	**< 0.001**	2182 (43.4%)	2188 (43.5%)	0.904
Unknown sex	360,916 (4.4%)	237 (4.7%)	0.300	243 (4.8%)	237 (4.7%)	0.779
**Race**						
White	5,594,554 (68.5%)	3566 (71.0%)	**< 0.001**	3311 (65.9%)	3566 (71.0%)	**< 0.001**
Unknown race	1,163,321 (14.2%)	642 (12.8%)	**0.003**	787 (15.7%)	642 (12.8%)	**< 0.001**
Black or African American	885,377 (10.8%)	478 (9.5%)	**0.003**	568 (11.3%)	478 (9.5%)	**0.003**
Other Race	223,761 (2.7%)	190 (3.8%)	**< 0.001**	170 (3.4%)	190 (3.8%)	0.283
Asian	259,488 (3.2%)	131 (2.6%)	**0.022**	156 (3.1%)	131 (2.6%)	0.134
Native Hawaiian or other Pacific Islander	27,614 (0.3%)	14 (0.3%)	0.469	16 (0.3%)	14 (0.3%)	0.715
American Indian or Alaska Native	18,976 (0.2%)	10 (0.2%)	0.625	17 (0.3%)	10 (0.2%)	0.177
**Ethnicity**						
Not Hispanic or Latino	5,781,038 (70.7%)	3415 (68.0%)	**< 0.001**	3468 (69.0%)	3415 (68.0%)	0.255
Hispanic or Latino	618,432 (7.6%)	633 (12.6%)	**< 0.001**	513 (10.2%)	633 (12.6%)	**< 0.001**
Unknown Ethnicity	1,773,621 (21.7%)	977 (19.4%)	**< 0.001**	1044 (20.8%)	977 (19.4%)	0.095
**Comorbidities and lifestyle factors**						
Congenital malformations of the circulatory system	84,154 (1.0%)	930 (18.5%)	**< 0.001**	73 (1.5%)	930 (18.5%)	**< 0.001**
Diabetes mellitus	919,234 (11.2%)	498 (9.9%)	**0.003**	300 (6.0%)	498 (9.9%)	**< 0.001**
Hypotension	181,276 (2.2%)	372 (7.4%)	**< 0.001**	87 (1.7%)	372 (7.4%)	**< 0.001**
Hypertensive diseases	2,190,297 (26.8%)	702 (14.0%)	**< 0.001**	694 (13.8%)	702 (14%)	0.818
Overweight and obesity	1,016,185 (12.4%)	1143 (22.7%)	**< 0.001**	551 (11.0%)	1143 (22.7%)	**< 0.001**
Alcohol use disorders	198,956 (2.4%)	25 (0.5%)	**< 0.001**	89 (1.8%)	25 (0.5%)	**< 0.001**
Tobacco use	199,476 (2.4%)	30 (0.6%)	**< 0.001**	115 (2.3%)	30 (0.6%)	**< 0.001**

*Note:* Data are represented as mean ± SD or *n* (%). DS, Down syndrome. All significant values are indicated in bold, *p* < 0.05.

^a^ Demographic parameters used to match groups.

### 3.2 Cancer Incidence

**FIGURE 2:**
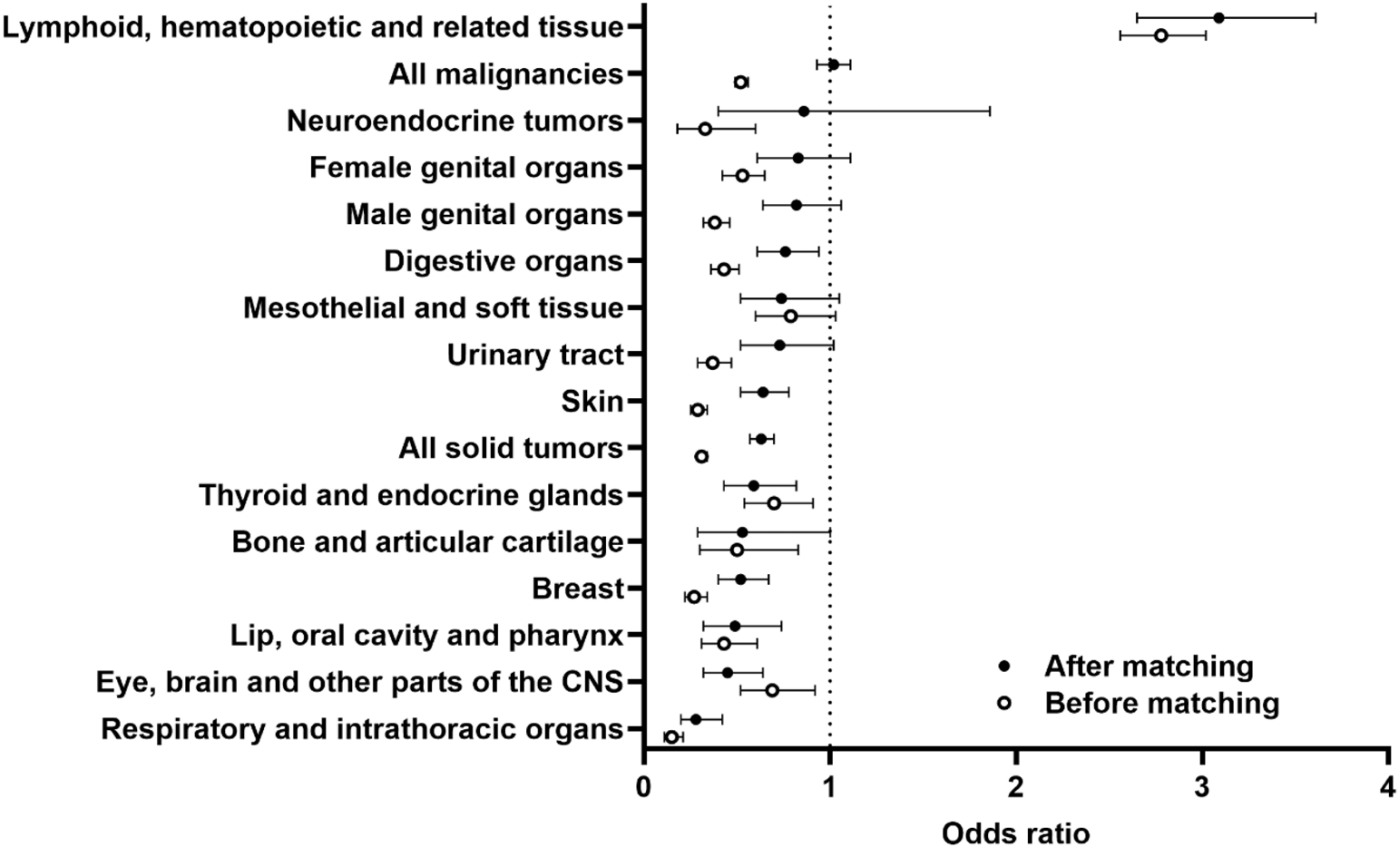
Cancer incidence in the primary organ systems before and after propensity score matching in individuals with and without DS, ≥ 15 years old. The data are represented as odds ratios with 95% confidence intervals comparing adults with DS to the control cohort. CNS, central nervous system.

After matching, all individuals with DS ≥ 15 years old were three times more likely to be diagnosed with malignancies of the lymphoid, hematopoietic, and related tissues (OR 3.09, CI 2.65–3.61, *p* < 0.001, Figure 2; Table S2). The likelihood of a diagnosis of any malignancy was not different between groups (OR 1.02, CI 0.93–1.11, *p* = 0.753); patients with DS were less likely to be diagnosed with any solid malignancy than the control group (OR 0.63, CI 0.57–0.7, *p* < 0.001). However, this decreased risk was not universally applicable to all solid tumor malignancies (Table S2).

Notably, in the matched cohorts, AYA DS patients were less likely to be diagnosed with Hodgkin lymphoma (OR 0.44, CI 0.22–0.89, *p* = 0.028), but were at an increased risk for acute lymphoid, myeloid, and chronic myeloid leukemias (Table 2). Surprisingly, adults ≥ 40 years were more likely to be diagnosed with myelodysplastic syndromes (OR 12.25, CI 6.41–23.42, *p* < 0.001) and myeloma (OR 1.66, CI 1.06–2.6, *p* = 0.026) and had an increased risk of all leukemias but not lymphoma. Testicular cancer (OR 2.73, CI 1.32–5.65, *p* = 0.005) was more likely to be diagnosed in individuals 40 years and older with DS, but they were less likely to be diagnosed with breast (OR 0.48, CI 0.36–0.63, *p* < 0.001) and prostate (OR 0.35, CI 0.24–0.53, *p* < 0.001) cancer [Table 3]. All individuals ≥ 15 years of age had a lower incidence of cervical (OR 0.52, CI 0.29–0.96, *p* = 0.044) and lung (OR 0.32, CI 0.2–0.51, *p* < 0.001) cancers, likely driven by decreased risk in patients ≥ 40 years old.

**TABLE 2:** The incidence of blood cancers across the lifespan of individuals with DS compared to those without DS.

	Before matching				After matching			
	Controls, *n* (%)	DS, *n* (%)	Odds ratio (95% CI)	*p*	Controls, *n* (%)	DS, *n* (%)	Odds ratio (95% CI)	*p*
**15–39 years**								
MDS	1609 (0.2%)	15 (0.7%)	4.25 (2.55–7.08)	**< 0.001**	10 (0.5%)	15 (0.7%)	1.52 (0.68–3.38)	0.324
AML	5549 (0.6%)	105 (5.3%)	9.38 (7.69–11.44)	**< 0.001**	13 (0.6%)	105 (5.3%)	8.90 (4.99–15.89)	**< 0.001**
ALL	7146 (0.8%)	163 (8.5%)	12.16 (10.34–14.30)	**< 0.001**	26 (1.3%)	163 (8.5%)	7.33 (4.82–11.15)	**< 0.001**
CML	2012 (0.2%)	12 (0.6%)	2.71 (1.53–4.78)	**0.002**	10 (0.5%)	12 (0.6%)	1.21 (0.52–2.81)	0.676
CLL	985 (0.1%)	23 (1.1%)	10.68 (7.05–16.19)	**< 0.001**	10 (0.5%)	23 (1.1%)	2.33 (1.11–4.90)	**0.023**
Myeloma	1359 (0.1%)	10 (0.5%)	3.32 (1.78–6.19)	**0.001**	10 (0.5%)	10 (0.5%)	1.00 (0.42–2.41)	1.000
Non‐Hodgkin lymphoma	10,373 (1.1%)	28 (1.3%)	1.22 (0.84–1.77)	0.293	25 (1.2%)	28 (1.3%)	1.12 (0.65–1.93)	0.783
Hodgkin lymphoma	8456 (0.9%)	11 (0.5%)	0.58 (0.32–1.06)	0.080	25 (1.2%)	11 (0.5%)	0.44 (0.22–0.89)	**0.028**
**40 years and older**								
MDS	49,604 (0.7%)	117 (4.7%)	6.81 (5.66–8.21)	**< 0.001**	10 (0.4%)	117 (4.7%)	12.25 (6.41–23.42)	**< 0.001**
AML	43,323 (0.6%)	164 (6.7%)	11.23 (9.58–13.16)	**< 0.001**	17 (0.7%)	164 (6.7%)	10.33 (6.25–17.08)	**< 0.001**
ALL	13,546 (0.2%)	44 (1.8%)	9.18 (6.81–12.38)	**< 0.001**	10 (0.4%)	44 (1.8%)	4.48 (2.25–8.93)	**< 0.001**
CML	18,871 (0.3%)	31 (1.3%)	4.59 (3.22–6.55)	**< 0.001**	10 (0.4%)	31 (1.3%)	3.13 (1.53–6.39)	**0.001**
CLL	56,516 (0.8%)	27 (1.1%)	1.33 (0.91–1.94)	0.176	10 (0.4%)	27 (1.1%)	2.72 (1.31–5.63)	**0.007**
Myeloma	83,999 (1.2%)	51 (2.1%)	1.70 (1.29–2.24)	**< 0.001**	31 (1.3%)	51 (2.1%)	1.66 (1.06–2.60)	**0.026**
Non‐Hodgkin lymphoma	125,518 (1.8%)	56 (2.3%)	1.24 (0.95–1.62)	0.127	39 (1.6%)	56 (2.3%)	1.45 (0.96–2.18)	0.097
Hodgkin lymphoma	21,297 (0.3%)	12 (0.5%)	1.56 (0.89–2.76)	0.169	10 (0.4%)	12 (0.5%)	1.20 (0.52–2.78)	0.831
**Combined ages (15 years and older)**								
MDS	51,580 (0.6%)	135 (2.7%)	4.37 (3.68–5.18)	**< 0.001**	12 (0.2%)	135 (2.7%)	11.57 (6.40–20.92)	**< 0.001**
AML	51,261 (0.6%)	287 (5.8%)	9.82 (8.71–11.07)	**< 0.001**	28 (0.6%)	287 (5.8%)	11.05 (7.48–16.32)	**< 0.001**
ALL	22,230 (0.3%)	223 (4.6%)	17.72 (15.48–20.28)	**< 0.001**	31 (0.6%)	223 (4.6%)	7.74 (5.31–11.30)	**< 0.001**
CML	22,252 (0.3%)	42 (0.8%)	3.10 (2.29–4.20)	**< 0.001**	14 (0.3%)	42 (0.8%)	3.03 (1.65–5.55)	**< 0.001**
CLL	57,897 (0.7%)	51 (1.0%)	1.44 (1.09–1.90)	**0.011**	18 (0.4%)	51 (1.0%)	2.86 (1.67–4.90)	**< 0.001**
Myeloma	86,485 (1.1%)	63 (1.3%)	1.19 (0.93–1.52)	0.198	27 (0.5%)	63 (1.3%)	2.35 (1.50–3.70)	**< 0.001**
Non‐Hodgkin lymphoma	141,695 (1.7%)	89 (1.8%)	1.02 (0.83–1.26)	0.872	65 (1.3%)	89 (1.8%)	1.37 (1.00–1.90)	0.062
Hodgkin lymphoma	33,810 (0.4%)	24 (0.5%)	1.16 (0.77–1.73)	0.548	43 (0.9%)	24 (0.5%)	0.56 (0.34–0.92)	**0.020**

*Note:* All significant values are indicated in bold, *p* < 0.05. Abbreviations: ALL, acute lymphoid leukemia; AML, acute myeloid leukemia; CLL, chronic lymphoid leukemia; CML, chronic myeloid leukemia; DS, down syndrome; MDS, myelodysplastic syndromes.

**TABLE 3:** The incidence of solid tumors in individuals with DS compared to individuals without DS grouped into AYA and non‐AYA patients.

	Before matching				After matching			
	Controls, *n* (%)	DS, *n* (%)	Odds ratio (95% CI)	*p*	Controls, *n* (%)	DS, *n* (%)	Odds ratio (95% CI)	*p*
**15–39 years**								
Testis	7805 (0.8%)	32 (1.5%)	1.86 (1.31–2.63)	**0.002**	26 (1.2%)	32 (1.5%)	1.23 (0.73–2.08)	0.509
Endometrium	2044 (0.2%)	10 (0.5%)	2.20 (1.18–4.11)	**0.029**	10 (0.5%)	10 (0.5%)	1.00 (0.42–2.41)	1.000
Colon	3833 (0.4%)	10 (0.5%)	1.17 (0.63–2.18)	0.603	10 (0.5%)	10 (0.5%)	1.00 (0.42–2.41)	1.000
Cervix	4725 (0.5%)	10 (0.5%)	0.95 (0.51–1.77)	1.000	10 (0.5%)	10 (0.5%)	1.00 (0.42–2.41)	1.000
Breast	10,578 (1.1%)	10 (0.5%)	0.42 (0.23–0.79)	**0.003**	17 (0.8%)	10 (0.5%)	0.59 (0.27–1.28)	0.246
Prostate	705 (0.1%)	10 (0.5%)	6.40 (3.42–11.96)	**< 0.001**	10 (0.5%)	10 (0.5%)	1.00 (0.42–2.41)	1.000
Lung	2749 (0.3%)	10 (0.5%)	1.64 (0.88–3.05)	0.149	10 (0.5%)	10 (0.5%)	1.00 (0.42–2.41)	1.000
**40 years and older**								
Testis	12,688 (0.2%)	27 (1.1%)	5.98 (4.09–8.74)	**< 0.001**	10 (0.4%)	27 (1.1%)	2.73 (1.32–5.65)	**0.005**
Endometrium	102,365 (1.5%)	25 (1.0%)	0.67 (0.45–1.00)	0.058	25 (1.0%)	25 (1.0%)	1.00 (0.57–1.75)	1.000
Colon	181,306 (2.6%)	36 (1.5%)	0.54 (0.39–0.76)	**< 0.001**	49 (2.0%)	36 (1.5%)	0.73 (0.47–1.13)	0.189
Cervix	44,072 (0.6%)	11 (0.4%)	0.69 (0.38–1.25)	0.268	18 (0.7%)	11 (0.4%)	0.61 (0.29–1.29)	0.264
Breast	506,879 (7.4%)	76 (3.1%)	0.40 (0.32–0.50)	**< 0.001**	154 (6.3%)	76 (3.1%)	0.48 (0.36–0.63)	**< 0.001**
Prostate	439,145 (6.4%)	33 (1.3%)	0.20 (0.14–0.28)	**< 0.001**	91 (3.7%)	33 (1.3%)	0.35 (0.24–0.53)	**< 0.001**
Lung	305,229 (4.5%)	20 (0.8%)	0.18 (0.11–0.27)	**< 0.001**	78 (3.2%)	20 (0.8%)	0.25 (0.15–0.41)	**< 0.001**
**Combined ages (15 years and older)**								
Testis	25,120 (0.3%)	82 (1.6%)	5.38 (4.33–6.70)	**< 0.001**	32 (0.6%)	82 (1.6%)	2.59 (1.72–3.9)	**< 0.001**
Endometrium	106,735 (1.3%)	29 (0.6%)	0.44 (0.31–0.63)	**< 0.001**	28 (0.6%)	29 (0.6%)	1.04 (0.62–1.74)	0.895
Colon	189,012 (2.3%)	49 (1.0%)	0.42 (0.31–0.55)	**< 0.001**	59 (1.2%)	49 (1.0%)	0.83 (0.57–1.21)	0.384
Cervix	55,479 (0.7%)	17 (0.3%)	0.50 (0.31–0.80)	**0.004**	32 (0.6%)	17 (0.3%)	0.53 (0.29–0.96)	**0.044**
Breast	532,575 (6.5%)	94 (1.9%)	0.27 (0.22–0.34)	**< 0.001**	179 (3.6%)	94 (1.9%)	0.52 (0.40–0.67)	**< 0.001**
Prostate	439,537 (5.4%)	38 (0.8%)	0.13 (0.10–0.18)	**< 0.001**	98 (2.0%)	38 (0.8%)	0.38 (0.26–0.56)	**< 0.001**
Lung	309,244 (3.8%)	24 (0.5%)	0.12 (0.08–0.18)	**< 0.001**	74 (1.5%)	24 (0.5%)	0.32 (0.20–0.51)	**< 0.001**

*Note:* All significant values are indicated in bold, *p* < 0.05. Abbreviations: AYA, adolescent and young adults; DS, Down syndrome.

### 3.3 Anthracycline Cardiotoxicity

**FIGURE 3:**
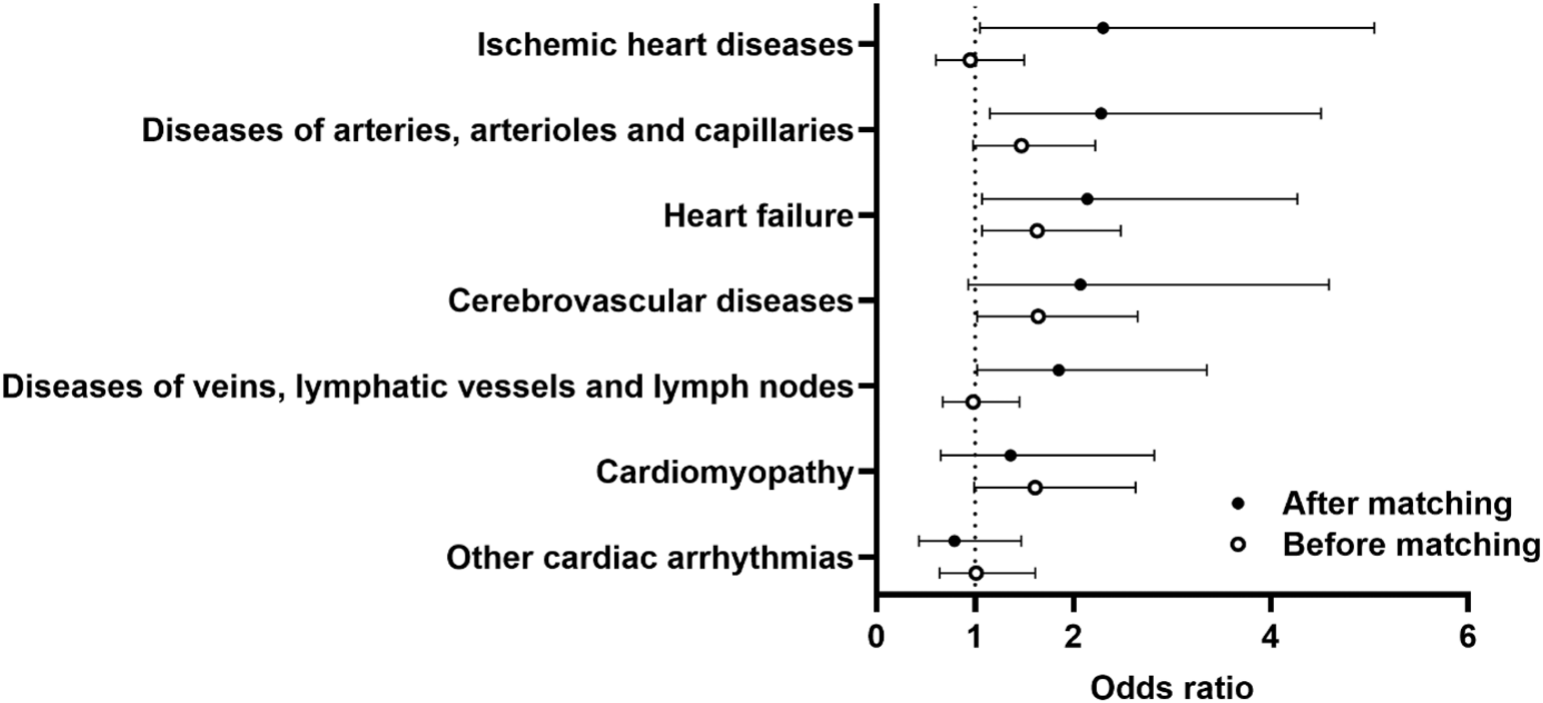
The incidence of cardiovascular diseases in patients with and without DS aged ≥ 15 years after treatment with anthracyclines. Data are represented as the odds ratios comparing adults with DS to the control group with 95% confidence intervals.

Because of the limited sample size, the incidence of cardiovascular disease after treatment with anthracycline medications was only evaluated for matched individuals ≥ 15 years old with and without DS. The average available follow‐up period was three years. The DS group had an increased rate of ischemic heart disease (OR 2.3, CI 1.05–5.05, *p* = 0.039), diseases of arteries, arterioles, and capillaries (OR 2.28, CI 1.15–4.51, *p* = 0.020), and heart failure (OR 2.14, CI 1.07–4.27, 0.042) (Table S4, Figure 3).

## 4 Discussion

We conducted a medical records review to assess the occurrence of hematological and solid malignancies in a large cohort of AYA and adults with DS. We found that individuals with DS are three times more likely to be diagnosed with malignancies of the lymphoid, hematopoietic, and related tissues compared to age and sex‐matched controls, such that the risk of blood cancer is not unique to the pediatric period. They also have a similar incidence of all malignancies but a lower or similar incidence of some solid tumors. We also report for the first time that their risk of myelodysplastic syndromes and multiple myeloma significantly increases after 40 years of age. Other heme malignancies, such as lymphoid and myeloid leukemia, are commonly diagnosed in both the AYA and non‐AYA cohorts with DS. The incidence of ischemic heart diseases, heart failure, and vascular diseases in individuals with DS and anthracycline medications is higher compared to the control group.

### 4.1 Hematological Malignancies

Altered hematopoiesis is one of the health conditions associated with DS, and we previously reported an increased risk of anemia in patients with DS (Deruisseau et al. 2023). It is well established that children with DS have a significantly increased risk of developing leukemia compared to children without DS (Baruchel et al. 2023). This has been associated with *GATA1* mutations, which primarily drive myeloid leukemia of DS (De Castro et al. 2021). Also, the overexpression of genes, including *DYRK1A*, *RCAN1*, *RUNX1*, *ERG*, and *HMGN1* on human chromosome 21, has been implicated in DS leukemogenesis (Zaslavsky et al. 2013; Rainis et al. 2005; Cabal‐Hierro et al. 2020). Like children with DS, our study shows that the risk of leukemia persists in adults. Kreig et al. found no significant difference in the risk of leukemia in adults ≥ 18 years old with DS compared to adults without DS (Krieg et al. 2024). However, Hasle et al. found a high risk of leukemia in young adults aged 10–29 years, with only three cases of leukemia in individuals > 30 years old (Hasle et al. 2000). The variability in the results may be due to the larger sample size in our study, which provided access to a broader population of adults with Down syndrome. Additional research is needed to investigate the mechanisms of leukemogenesis in AYA and older adult patients with DS, which will improve treatment options for this emerging population. It is essential to acknowledge that some cases of leukemia in our adult population could be due to relapses from childhood leukemia since patients with DS and acute lymphoid leukemia have a higher risk of relapse compared to patients without DS (Michels et al. 2021). However, we excluded any neoplasm that occurred before the age of 15 years for all patients to attempt to minimize this confounder.

Myelodysplastic syndromes arise from impaired hematopoietic differentiation (Hitzler and Zipursky 2005) and are a risk factor for progression to leukemia. One case report detailing an adult with DS described myelodysplastic syndromes as a rare occurrence in the adult DS population (Virk and Naseem 2020). However, we observed that individuals aged 40 years and older with DS were more likely to be diagnosed with myelodysplastic syndromes compared to individuals without DS, highlighting the power of our large sample size to identify more rare hematological conditions. Myelodysplastic syndromes are a risk factor for hematological malignancies, but because data are reported in aggregate, we were unable to discern the degree to which myelodysplastic syndromes precede the development of hematological malignancies in our study.

Adults aged 40 and older with DS had a higher incidence of multiple myeloma compared to those without DS. Hasle et al. found no multiple myeloma cases in their study (Hasle et al. 2016). However, our study consisted of a larger group of individuals with DS and sufficient power to quantify rare malignancies. A single case report on multiple myeloma in an adult with DS identified it as a rare incident with no established treatment protocols and treatment complications (Musleh and Alhusein 2024). Given that multiple myeloma is associated with aging, with a median age of 69 at diagnosis in the general population, our data suggest that the DS population is gradually developing previously underappreciated age‐related malignancies (Greteman et al. 2024). Therefore, creating clearer therapeutic guidelines for multiple myeloma in the adult DS population may be necessary. In this study, the age at index of neoplasms for patients with DS was significantly lower compared to patients without DS. This could be due to the accelerated aging associated with DS (Zigman 2013). More studies are needed to determine the factors increasing the predisposition of adults with DS to these hematological malignancies, including studies of clonal hematopoiesis and obesity as a potential risk factor in DS where the incidence is higher (Carson et al. 2014). That said, while obesity is a risk factor for multiple myeloma, obese patients are better able to tolerate aggressive treatment regimens (Beason et al. 2013). It is not known whether this translates to DS. It is also unknown if the driving factor for the increased incidence of myeloma is a higher rate of monoclonal gammopathy of undetermined significance (MGUS), a pre‐malignant condition, as observed in individuals of African descent, or an increased rate of progression from MGUS to multiple myeloma (Tomasson et al. 2018). Future studies should focus on addressing these unknowns in DS, including guidelines to aid in the prevention and early detection of hematological malignancies.

### 4.2 Solid Tumors

Consistent with previous reports, we found a similar prevalence of many malignancies and a lower prevalence of certain solid tumors in our matched cohorts (Patja et al. 2006; Krieg et al. 2024; Hasle et al. 2016). Upon classifying these malignancies into specific subgroups, we observed a higher incidence of testicular cancer in adults (≥ 40 years old) with DS. We found a similar incidence of testicular cancer in AYA with and without DS. Combining all age groups, others also observed an increased incidence of testicular cancer (Patja et al. 2006; Hasle et al. 2016). Individuals with DS have a high incidence of cryptorchidism, which is a risk factor for seminoma, a subtype of testicular cancer (Benson Jr. et al. 1991). The authors of a case report on seminoma in an adult with DS recommended that individuals with DS aged 15 to 45 undergo annual testicular examinations (Hengy et al. 2022). The ≥ 40‐year‐old cohort with DS had a significantly lower incidence of breast and prostate cancer, which was persistent when both groups were combined, and a lower incidence of skin and cervical cancer when the groups were combined.

The lower occurrence of some solid tumors in the DS population could be due to several factors, including the gene dosage of tumor suppressor genes on human chromosome 21, increased apoptosis, and DNA damage associated with DS (Osuna‐Marco et al. 2021). The reduced incidence of solid tumors in DS may be attributed to the inhibition of VEGF‐mediated angiogenesis due to the overexpression of *DSCR1* and *DYRK1a* on human chromosome 21 (Baek et al. 2009). Environmental factors likely also play a role. Compared to the cohort without DS, individuals with DS had less documented exposure to environmental risk factors for cancer development, such as alcohol‐related disorders and tobacco use. While the degree to which this is consistently documented in the medical record is not clear, it offers an intriguing environmental hypothesis for cancer risk. Recently, alcohol use has been documented as an important risk factor for breast and prostate cancer (Teissedre et al. 2020). It has been suggested that women with DS experience early menopause, which may reduce their risk of breast cancer (Seltzer et al. 2001). It is essential to note that while adults with DS may have a lower incidence of certain solid tumors, they are not protected from these conditions. Hence, regular screening remains necessary. Additionally, they share a similar risk for some cancers and should undergo the same routine screenings as the general population.

### 4.3 Cardiotoxicity

Anthracycline‐based regimens are broadly used to treat several cancer types (Hefti and Blanco 2016; O'Brien et al. 2008). Some underlying risk factors of cardiotoxicity in pediatric patients after anthracycline exposure include age, type of treatment, sex, genetic factors such as DS, cumulative dose, and pre‐existing cardiovascular disease (Krischer et al. 1997; Venegas‐Zamora et al. 2021). After observing a higher incidence of some clinical manifestations of anthracycline cardiotoxicity, such as heart failure and ischemic heart diseases, we explored the DS population to investigate whether our observations were due to our previously documented increased overall risk of cardiovascular disease in DS (Bates et al. 2023) (Table S6). We found that even within the DS population, there was still an increased risk of cardiovascular disease with anthracycline treatment. Our findings in the DS population were compared to those of individuals without DS, where we also found an increased risk (Table S8). Therefore, even though anthracyclines lead to cardiovascular toxicity generally, this risk is increased in adults with DS and may be enhanced by their inherent cardiovascular disease risk. The overexpression of *DRYK1A* and *RCAN1* inhibits the calcineurin/NFAT pathway, which is essential for cardiovascular development and activation of endothelial cells during angiogenesis (Laham et al. 2021; Minami 2014). Reduced angiogenesis leads to endothelial dysfunction (Laham et al. 2021). Anthracyclines also lead to vascular endothelial dysfunction (Clayton et al. 2020). Thus, anthracycline treatment could exacerbate the already existing endothelial dysfunction associated with DS (Cappelli‐Bigazzi et al. 2004), further increasing their risk of cardiovascular diseases. TNF‐κB is a transcription factor involved in immune cell regulation and inflammation (Dabbah‐Krancher and Snow 2023) and is impaired in DS, partly due to increased cytosolic levels of the NF‐κB inhibitor, which can lead to impaired lymphocyte responses (Dabbah‐Krancher and Snow 2023; Granese et al. 2013). This can lead to prolonged inflammation, myocardial and vascular damage, and impede tissue recovery (Wang et al. 2025). The increased incidence of cardiovascular diseases in our research serves as a basis to further mechanistically investigate cardiovascular complications in adults with DS.

Finally, as noted in our earlier study, individuals with DS experience a higher incidence of cardiovascular diseases compared to age‐ and sex‐matched peers without DS (Bates et al. 2023). This suggests that pre‐existing increased cardiovascular risk in DS may exacerbate the effects of cardiotoxic therapy in this population. Patients with DS were more likely to be hypotensive and hypertensive prior to cancer diagnoses. This emphasizes the necessity for mechanistically driven studies and organized and individualized care for this unique population.

### 4.4 Strengths and Limitations

An essential feature of this study is the large cohort of adults with DS that allows for increased power to detect rarer cancers, including multiple myeloma. However, the limitations of this study include our inability to access individual patient records and perform case–control matching based on the date of diagnosis. As a result, we could not identify the dose of anthracyclines for each group. Due to the retrospective nature of this study, the data show association and not causality. Using ICD‐10 codes is subject to miscoding and nonspecific diagnoses. We also do not know how many patients had multiple neoplasm diagnoses, so we did not correct for possible multiple comparisons. Lastly, some healthcare centers for individuals with disabilities may not be part of the TriNetX network, resulting in missed data from those centers, although our data set represents a diverse geographic area and includes most of the large centers that provide comprehensive cancer and developmental care. Because patients cannot be compared between geographic regions, we do not know whether risk differences are geographically driven.

## 5 Conclusions

We found that individuals with DS are more likely to develop cardiovascular diseases after treatment with anthracyclines and more likely to develop age‐related hematological malignancies than previously reported. Diseases like multiple myeloma, considered rare in individuals with DS, should be reexamined as life expectancy continues to rise. Despite the low incidence of solid tumors in this group, they are not entirely protected from these malignancies and are at similar risk for some. It is essential to consider appropriate screening methods and timing for adults with DS as they age. Given the differences between our matched and unmatched cohorts for some cancers, DS individuals may experience these tumors earlier than typically seen in the general population. The absence of established healthcare guidelines for AYA and adult DS patients may result in lower survival rates, potentially exclude them from important clinical studies, and reduce life expectancy. Given the increased risk of cardiovascular disease in DS individuals, increased screening following cardiotoxic chemotherapy may be warranted. Studies examining clonal hematopoiesis and the effects of aging on bone marrow function in adults with DS are warranted. Our limited understanding, along with the results from this extensive real‐world cohort study, underlines the need for future cancer screening and survivorship guidelines to be tailored to the specific needs of the aging DS population.

## Author Contributions

M.A.B., A.V., C.R.J., H.K., M.V., D.S.D., M.H.T., and M.L.B.—participated in the conceptualization, method design, data collection and analysis, interpretation of results, figure and manuscript preparation, and gave approval of the final version.

## Disclosure

Dr. Bates is the Founder and CEO of LSF Medical Solutions and Dr. Tomasson serves as Chief Medical Officer. Their work at LSF Medical Solutions does not overlap topically with the content of this manuscript.

## Conflicts of Interest

Dr. Bates is the Founder and CEO of LSF Medical Solutions and Dr. Tomasson serves as Chief Medical Officer. Their work at LSF Medical Solutions does not overlap topically with the content of this manuscript.

## Supporting information


**Data S1:** cph470037‐sup‐0001‐Tables.docx.

## Data Availability

The data that support the findings of this study are available on request from the corresponding author. The data are not publicly available due to privacy or ethical restrictions.
