# Supplementary material for: Cancer and Post‐Therapy Cardiotoxicity Risk in Adolescents, Young Adults, and Adults With Down Syndrome
Source: Compr Physiol. 2025 Sep 5;15(5):e70037. doi: 10.1002/cph4.70037 (PMC12413507; doi:10.1002/cph4.70037)
Supplement: Supplementary file 1 — Data S1: cph470037‐sup‐0001‐Tables.docx. [file CPH4-15-e70037-s001.docx]

| Supplemental Table 1. Diagnosis and procedure codes used for TriNetX search. | | | |
| --- | --- | --- | --- |
| **Procedure Code** | **Diagnosis** | **Procedure Code** | **Diagnosis** |
| CPT:1013626 | Office or Other Outpatient Services | ICD10CM:C81-C96 | Malignant neoplasm of lymphoid, hematopoietic and related tissue |
| CPT:1013659 | Hospital Inpatient and Observation Care Services | ICD10CM:18 | Malignant neoplasm of colon |
| ICD10CM:Q90 | Down syndrome | ICD10CM:C34 | Malignant neoplasm of lung |
| ICD10CM:C00-D49 | Neoplasms | ICD10CM:C53 | Malignant neoplasm of cervix uteri |
| ICD10CM:E08-E13 | Diabetes mellitus | ICD10CM:C54.1 | Malignant neoplasm of endometrium |
| ICD10CM:E66 | Overweight and obesity | ICD10CM:C61 | Malignant neoplasm of prostate |
| ICD10CM:F10 | Alcohol-related disorders | ICD10CM:C62 | Malignant neoplasm of testis |
| ICD10CM:I10-I1A | Hypertensive diseases | ICD10CM:C81 | Hodgkin lymphoma |
| ICD10CM:I95 | Hypotension | ICD10CM:C85 | Non-Hodgkin lymphoma |
| ICD10CM:Q20-Q28 | Congenital malformations of the circulatory system | ICD10CM:C90.0 | Multiple myeloma |
| ICD10CM:Z72.0 | Tobacco use | ICD10CM:C91.0 | Acute lymphoblastic leukemia |
| ICD10CM:C00-C14 | Malignant neoplasm of lip, oral cavity and pharynx | ICD10CM:91.1 | Chronic lymphoid leukemia |
| ICD10CM:C15-C26 | Malignant neoplasm of digestive organs | ICD10CM:C92.1, C92.2 | Chronic myeloid leukemia |
| ICD10CM:C30-C39 | Malignant neoplasm of respiratory and intrathoracic organs | ICD10CM:C92.0, C92.4, C92.5, C92.6, C93.0, C95.0, C94.2 | Acute myeloid leukemia |
| ICD10CM:C40-C41 | Malignant neoplasm of bone and articular cartilage | ICD10CM:D46 | Myelodysplastic syndromes |
| ICD10CM:C43-44 | Malignant neoplasm of skin | ATC:L01DB | Anthracyclines and related substances |
| ICD10CM:C45-C49 | Malignant neoplasm of mesothelial and soft tissue | ICD10CM:I20-I25 | Ischemic heart diseases |
| ICD10CM:C50 | Malignant neoplasm of breast | ICD10CM:I42 | Cardiomyopathy |
| ICD10CM:C51-C58 | Malignant neoplasm of female genital organs | ICD10CM:I49 | Other cardiac arrhythmias |
| ICD10CM:C60-63 | Malignant neoplasm of male genital organs | ICD10CM:I50 | Heart failure |
| ICD10CM:C64-C68 | Malignant neoplasm of urinary tract | ICD10CM:I63-67 | Cerebrovascular diseases |
| ICD10CM:C69-C72 | Malignant neoplasm of eye, brain and other parts of CNS | ICD10CM:I70-I79 | Diseases of arteries, arterioles and capillaries |
| ICD10CM:C73-C75 | Malignant neoplasm of thyroid and endocrine glands | ICD10CM:I80-I89 | Diseases of veins, lymphatic vessels and lymph nodes |
| ICD10CM:C7A-C7A | Malignant neuroendocrine tumors |  |  |
| CPT, Current Procedural Terminology; ICD10CM, International Classification of Diseases, Tenth Revision, Clinical Modification; ATC, Anatomical Therapeutic Chemical; TNX, TriNetX curated; CNS, Central nervous system. | | | |

| Supplemental Table 2. The incidence of cancer in the major body sites before and after matching. | | | | | | | | |
| --- | --- | --- | --- | --- | --- | --- | --- | --- |
|  | **Before matching** | | | | **After matching** | | | |
|  | **Controls, n (%)** | **DS, n (%)** | **Odds ratio (95% CI)** | **p-value** | **Controls, n (%)** | **DS, n (%)** | **Odds ratio (95% CI)** | **p-value** |
| Lymphoid, hematopoietic and related tissue | 432,373 (5.3%) | 639 (13.5%) | 2.78 (2.56-3.02) | **<0.001** | 238 (4.8%) | 639 (13.4%) | 3.09 (2.65-3.61) | **<0.001** |
| All malignancies | 3,559,705 (43.8%) | 1,364 (28.9%) | 0.52 (0.49-0.56) | **<0.001** | 1,408 (28.6%) | 1,366 (28.9%) | 1.02 (0.93-1.11) | 0.753 |
| Neuroendocrine tumors | 53,630 (0.7%) | 11 (0.2%) | 0.33 (0.18-0.60) | **<0.001** | 14 (0.3%) | 12 (0.2%) | 0.86 (0.4-1.86) | 0.845 |
| Female genital organs | 250,154 (3.1%) | 82 (1.6%) | 0.53 (0.42-0.65) | **<0.001** | 99 (2.0%) | 82 (1.6%) | 0.83 (0.61-1.11) | 0.230 |
| Male genital organs | 467,774 (5.7%) | 114 (2.3%) | 0.38 (0.32-0.46) | **<0.001** | 138 (2.7%) | 114 (2.3%) | 0.82 (0.64-1.06) | 0.142 |
| Digestive organs | 528,917 (6.5%) | 145 (2.9%) | 0.43 (0.36-0.51) | **<0.001** | 190 (3.8%) | 145 (2.9%) | 0.76 (0.61-0.94) | **0.014** |
| Mesothelial and soft tissue | 110,961 (1.4%) | 54 (1.1%) | 0.79 (0.60-1.03) | 0.096 | 73 (1.5%) | 54 (1.1%) | 0.74 (0.52-1.05) | 0.091 |
| Urinary tract | 260,107 (3.2%) | 60 (1.2%) | 0.37 (0.29-0.47) | **<0.001** | 82 (1.6%) | 60 (1.2%) | 0.73 (0.52-1.02) | 0.064 |
| Skin | 849,868 (10.4%) | 163 (3.2%) | 0.29 (0.25-0.34) | **<0.001** | 252 (5.0%) | 164 (3.3%) | 0.64 (0.52-0.78) | **<0.001** |
| All solid tumors | 3,277,350 (40.3%) | 862 (17.4%) | 0.31 (0.29-0.34) | **<0.001** | 1,242 (25.0%) | 864 (17.4%) | 0.63 (0.57-0.70) | **<0.001** |
| Thyroid and endocrine glands | 134,438 (1.6%) | 58 (1.2%) | 0.70 (0.54-0.91) | **0.008** | 97 (1.9%) | 58 (1.2%) | 0.59 (0.43-0.82) | **0.002** |
| Bone and articular cartilage | 48,908 (0.6%) | 15 (0.3%) | 0.50 (0.30-0.83) | **0.008** | 28 (0.6%) | 15 (0.3%) | 0.53 (0.29-1.00) | **0.048** |
| Breast | 532,575 (6.5%) | 94 (1.9%) | 0.27 (0.22-0.34) | **<0.001** | 179 (3.6%) | 94 (1.9%) | 0.52 (0.40-0.67) | **<0.001** |
| Lip, oral cavity and pharynx | 122,792 (1.5%) | 33 (0.7%) | 0.43 (0.31-0.61) | **<0.001** | 67 (1.3%) | 33 (0.7%) | 0.49 (0.32-0.74) | **0.001** |
| Eye, brain and other parts of the CNS | 110,802 (1.4%) | 47 (0.9%) | 0.69 (0.52-0.92) | **0.012** | 102 (2.0%) | 47 (0.9%) | 0.45 (0.32-0.64) | **<0.001** |
| Respiratory and intrathoracic organs | 362,029 (4.4%) | 35 (0.7%) | 0.15 (0.11-0.21) | **<0.001** | 121 (2.4%) | 35 (0.7%) | 0.28 (0.20-0.42) | **<0.001** |
| DS, Down syndrome; CNS, central nervous system. All significant odds ratios are indicated in bold, p<0.05. | | | | | | | | |

| Supplemental Table 3. Baseline characteristics of patients with and without DS after treatment with anthracyclines. | | | | | | |
| --- | --- | --- | --- | --- | --- | --- |
|  | **Before matching** | | | **After matching** | | |
|  | **Controls**  **n=102,976** | **DS**  **n=177** | **p-value** | **Controls**  **n=175** | **DS**  **n=175** | **p-value** |
| **Age, years** | | | | | | |
| ^†^Age at index | 55.2 ± 15.9 | 41.7 ± 18.6 | **< 0.001** | 41.7 ± 18.6 | 41.7 ± 18.6 | 1.000 |
| Current age | 62.1 ± 16.1 | 48.6 ± 18.9 | **< 0.001** | 48.7 ± 19.3 | 48.6 ± 18.9 | 0.940 |
| **Sex** | | | | | | |
| ^†^Female | 58,704 (58.8%) | 77 (44%) | **< 0.001** | 77 (44.0%) | 77 (44.0%) | 1.000 |
| Male | 38,025 (38.1%) | 94 (53.7%) | **< 0.001** | 92 (52.6%) | 94 (53.7%) | 0.830 |
| Unknown sex | 3,064 (3.1%) | 10 (5.7%) | **0.043** | 10 (5.7%) | 10 (5.7%) | 1.000 |
| **Race** | | | | | | |
| White | 68,302 (68.4%) | 115 (65.7%) | 0.438 | 120 (68.6%) | 115 (65.7%) | 0.569 |
| Unknown race | 10,621 (10.6%) | 13 (7.4%) | 0.168 | 22 (12.6%) | 13 (7.4%) | 0.109 |
| Black or African American | 11,841 (11.9%) | 19 (10.9%) | 0.680 | 16 (9.1%) | 19 (10.9%) | 0.593 |
| Other Race | 3,751 (3.8%) | 17 (9.7%) | **< 0.001** | 10 (5.7%) | 17 (9.7%) | 0.161 |
| Asian | 4,344 (4.4%) | 10 (5.7%) | 0.378 | 11 (6.3%) | 10 (5.7%) | 0.822 |
| Native Hawaiian or other Pacific Islander | 621 (0.6%) | 10 (5.7%) | **< 0.001** | 0 | 10 (5.7%) | **0.001** |
| American Indian or Alaska Native | 313 (0.3%) | 10 (5.7%) | **< 0.001** | 0 | 10 (5.7%) | **0.001** |
| **Ethnicity** | | | | | | |
| Not Hispanic or Latino | 72,356 (72.5%) | 118 (67.4%) | 0.133 | 118 (67.4%) | 118 (67.4%) | 1.000 |
| Hispanic or Latino | 8,309 (8.3%) | 35 (20.0%) | **< 0.001** | 19 (10.9%) | 35 (20.0%) | **0.018** |
| Unknown Ethnicity | 19,128 (19.2%) | 22 (12.6%) | **0.027** | 38 (21.7%) | 22 (12.6%) | **0.023** |
| **Comorbidities and lifestyle factors** | | | | | | |
| Congenital malformations of the circulatory system | 1,856 (1.9%) | 23 (13.1%) | **< 0.001** | 10 (5.7%) | 23 (13.1%) | **0.017** |
| Diabetes mellitus | 17,839 (17.9%) | 33 (18.9%) | 0.735 | 23 (13.1%) | 33 (18.9%) | 0.145 |
| Hypotension | 9,167 (9.2%) | 25 (14.3%) | **0.020** | 22 (12.6%) | 25 (14.3%) | 0.638 |
| Hypertensive diseases | 42,174 (42.3%) | 66 (37.7%) | 0.224 | 47 (26.9%) | 66 (37.7%) | **0.030** |
| Overweight and obesity | 19,736 (19.8%) | 45 (25.7%) | 0.049 | 28 (16.0%) | 45 (25.7%) | **0.025** |
| Data are represented as mean ± SD or n (%). DS, Down syndrome. All significant values are indicated in bold, p<0.05. ^†^Demographic parameters used to match groups. | | | | | | |

| Supplemental Table 4. The incidence of cardiovascular diseases in adults with and without DS after treatment with anthracyclines. | | | | | | | | |
| --- | --- | --- | --- | --- | --- | --- | --- | --- |
|  | **Before matching** | | | | **After matching** | | | |
|  | **Controls, n (%)** | **DS, n (%)** | **Odds ratio (95% CI)** | **p-value** | **Controls, n (%)** | **DS, n (%)** | **Odds ratio (95% CI)** | **p-value** |
| Ischemic heart diseases | 11,988 (13.7%) | 21 (13.1%) | 0.95 (0.60-1.50) | 0.909 | 10 (6.2%) | 21 (13.1%) | 2.30 (1.05-5.05) | **0.039** |
| Diseases of arteries, arterioles and capillaries | 11,366 (12.8%) | 28 (17.7%) | 1.47 (0.98-2.22) | 0.073 | 14 (8.6%) | 28 (17.7%) | 2.28 (1.15-4.51) | **0.020** |
| Heart failure | 10,195 (10.9%) | 26 (16.6%) | 1.63 (1.07-2.48) | **0.029** | 14 (8.5%) | 26 (16.6%) | 2.14 (1.07-4.27) | **0.042** |
| Cerebrovascular diseases | 6,811 (7.2%) | 19 (11.3%) | 1.64 (1.02-2.65) | 0.050 | 10 (5.8%) | 19 (11.3%) | 2.07 (0.93-4.59) | 0.082 |
| Diseases of veins, lymphatic vessels and lymph nodes | 20,892 (26.2%) | 35 (25.9%) | 0.98 (0.67-1.45) | 1.000 | 22 (15.9%) | 35 (25.9%) | 1.85 (1.02-3.35) | 0.053 |
| Cardiomyopathy | 6,716 (7.0%) | 18 (10.8%) | 1.61 (0.99-2.63) | 0.066 | 14 (8.2%) | 18 (10.8%) | 1.36 (0.65-2.82) | 0.461 |
| Other cardiac arrhythmias | 12,482 (13.9%) | 21 (14.1%) | 1.01 (0.64-1.61) | 0.906 | 27 (17.2%) | 21 (14.1%) | 0.79 (0.43-1.47) | 0.530 |
| DS, Down syndrome. All significant values are indicated in bold, p<0.05. | | | | | | | | |

| Supplemental Table 5. Baseline characteristics with Down syndrome. | | | | | | |  |  |  |  |  |  |
| --- | --- | --- | --- | --- | --- | --- | --- | --- | --- | --- | --- | --- |
|  | **Before matching** | | | **After matching** | | |  |  |  |  |  |  |
|  | **No anthracyclines**  **n=35,229** | **Anthracyclines**  **n=177** | **p-value** | **DS-no anthracyclines**  **n=175** | **DS**  **n=175** | **p-value** |  |  |  |  |  |  |
| **Age, years** | | | | | | |  |  |  |  |  |  |
| ^†^Age at index | 24.1 ± 18.0 | 41.7 ± 18.6 | **< 0.001** | 41.7 ± 18.6 | 41.7 ± 18.6 | 1.000 |  |  |  |  |  |  |
| Current age | 33.8 ± 16.5 | 48.6 ± 18.9 | **< 0.001** | 50.3 ± 19.3 | 48.6 ± 18.9 | 0.393 |  |  |  |  |  |  |
| **Sex** | | | | | | |  |  |  |  |  |  |
| ^†^Female | 15,332 (48.9%) | 77 (44.0%) | 0.192 | 77 (44.0%) | 77 (44.0%) | 1.000 |  |  |  |  |  |  |
| Male | 15,283 (48.8%) | 94 (53.7%) | 0.193 | 92 (52.6%) | 94 (53.7%) | 0.830 |  |  |  |  |  |  |
| Unknown sex | 713 (2.3%) | 10 (5.7%) | **0.003** | 10 (5.7%) | 10 (5.7%) | 1.000 |  |  |  |  |  |  |
| **Race** | | | | | | |  |  |  |  |  |  |
| White | 20,783 (66.3%) | 115 (65.7%) | 0.861 | 119 (68.0%) | 115 (65.7%) | 0.650 |  |  |  |  |  |  |
| Unknown race | 4,745 (15.1%) | 13 (7.4%) | **0.005** | 25 (14.3%) | 13 (7.4%) | **0.039** |  |  |  |  |  |  |
| Black or African American | 3,056 (9.8%) | 19 (10.9%) | 0.624 | 12 (6.9%) | 19 (10.9%) | 0.188 |  |  |  |  |  |  |
| Other Race | 1,758 (5.6%) | 17 (9.7%) | **0.019** | 12 (6.9%) | 17 (9.7%) | 0.332 |  |  |  |  |  |  |
| Asian | 828 (2.6%) | 10 (5.7%) | **0.012** | 10 (5.7%) | 10 (5.7%) | 1.000 |  |  |  |  |  |  |
| Native Hawaiian or other Pacific Islander | 70 (0.2%) | 10 (5.7%) | **< 0.001** | 10 (5.7%) | 10 (5.7%) | 1.000 |  |  |  |  |  |  |
| American Indian or Alaska Native | 88 (0.3%) | 10 (5.7%) | **< 0.001** | 0 | 10 (5.7%) | **0.001** |  |  |  |  |  |  |
| **Ethnicity** | | | | | | |  |  |  |  |  |  |
| Not Hispanic or Latino | 19,826 (63.3%) | 118 (67.4%) | 0.257 | 108 (61.7%) | 118 (67.4%) | 0.264 |  |  |  |  |  |  |
| Hispanic or Latino | 5,060 (16.2%) | 35 (20.0%) | 0.168 | 24 (13.7%) | 35 (20.0%) | 0.116 |  |  |  |  |  |  |
| Unknown Ethnicity | 6,442 (20.6%) | 22 (12.6%) | **0.009** | 43 (24.6%) | 22 (12.6%) | **0.004** |  |  |  |  |  |  |
| **Comorbidities and lifestyle factors** | | | | | | |  |  |  |  |  |  |
| Congenital malformations of the circulatory system | 2,280 (7.3%) | 23 (13.1%) | **0.003** | 10 (5.7%) | 23 (13.1%) | **0.017** |  |  |  |  |  |  |
| Diabetes mellitus | 725 (2.3%) | 33 (18.9%) | **< 0.001** | 10 (5.7%) | 33 (18.9%) | **< 0.001** |  |  |  |  |  |  |
| Hypotension | 237 (0.8%) | 25 (14.3%) | **< 0.001** | 10 (5.7%) | 25 (14.3%) | **0.008** |  |  |  |  |  |  |
| Hypertensive diseases | 726 (2.3%) | 66 (37.7%) | **< 0.001** | 10 (5.7%) | 66 (37.7%) | **< 0.001** |  |  |  |  |  |  |
| Overweight and obesity | 1,033 (3.3%) | 45 (25.7%) | **< 0.001** | 10 (5.7%) | 45 (25.7%) | **< 0.001** |  |  |  |  |  |  |
| Data are represented as mean ± SD or n (%). DS, Down syndrome. All significant values are indicated in bold, p<0.05. ^†^Demographic parameters used to match groups. | | | | | | |  |  |  |  |  |  |

| Supplemental Table 6. The incidence of cardiovascular diseases in adults with Down syndrome comparing those with and without anthracyclines. | | | | | | | | |
| --- | --- | --- | --- | --- | --- | --- | --- | --- |
|  | **Before matching** | | | | **After matching** | | | |
|  | **No anthracyclines, n (%)** | **Anthracyclines, n (%)** | **Odds ratio (95% CI)** | **p-value** | **No anthracyclines, n (%)** | **Anthracyclines, n (%)** | **Odds ratio (95% CI)** | **p-value** |
| Ischemic heart diseases | 605 (2.0%) | 21 (13.1%) | 7.60 (4.77-12.1) | **<0.001** | 10 (6.0%) | 21 (13.1%) | 2.36 (1.07-5.18) | **0.037** |
| Diseases of arteries, arterioles and capillaries | 918 (3.0%) | 28 (17.7%) | 7.06 (4.67-10.67) | **<0.001** | 10 (5.9%) | 28 (17.7%) | 3.45 (1.61-7.36) | **0.001** |
| Heart failure | 1,113 (3.6%) | 26 (16.6%) | 5.28 (3.45-8.07) | **<0.001** | 10 (6.1%) | 26 (16.6%) | 3.08 (1.43-6.61) | **0.004** |
| Cerebrovascular diseases | 658 (2.1%) | 19 (11.3%) | 5.85 (3.60-9.48) | **<0.001** | 10 (6.0%) | 19 (11.3%) | 2.02 (0.91-4.47) | 0.119 |
| Diseases of veins, lymphatic vessels and lymph nodes | 1,286 (4.2%) | 35 (25.9%) | 8.01 (5.43-11.82) | **<0.001** | 10 (6.0%) | 35 (25.9%) | 5.50 (2.61-11.59) | **<0.001** |
| Cardiomyopathy | 316 (1.0%) | 18 (10.8%) | 11.87 (7.19-19.61) | **<0.001** | 10 (5.9%) | 18 (10.8%) | 1.95 (0.87-4.35) | 0.116 |
| Other cardiac arrhythmias | 1,998 (6.6%) | 21 (14.1%) | 2.32 (1.46-3.69) | **0.001** | 10 (5.8%) | 21 (14.1%) | 2.67 (1.22-5.88) | **0.014** |
| DS, Down syndrome. All significant values are indicated in bold, p<0.05. | | | | | | | | |

| Supplemental Table 7. Baseline characteristics of patients without DS. | | | | | | |
| --- | --- | --- | --- | --- | --- | --- |
|  | **Before matching** | | | **After matching** | | |
|  | **No Ds and anthracyclines**  **n=31,145,476** | **No DS and no anthracyclines**  **n=102,976** | **p-value** | **Controls**  **n=99,790** | **DS**  **n=99,790** | **p-value** |
| **Age, years** | | | | | | |
| ^†^Age at index | 40.6 ± 22.1 | 55.2 ± 15.9 | **< 0.001** | 55.2 ± 15.9 | 55.2 ± 15.9 | 1.000 |
| Current age | 47.7 ± 21.5 | 62.1 ± 16.1 | **< 0.001** | 61.9 ± 16.3 | 62.1 ± 16.1 | 0.090 |
| **Sex** | | | | | | |
| ^†^Female | 15,730,104 (52.7%) | 58,704 (58.8%) | **< 0.001** | 58,704 (58.8%) | 58,704 (58.8%) | 1.000 |
| Male | 13,135,405 (44.0%) | 38,022 (38.1%) | **< 0.001** | 37,931 (38.0%) | 38,022 (38.1%) | 0.675 |
| Unknown sex | 955,744 (3.2%) | 3,064 (3.1%) | **0.016** | 3,155 (3.2%) | 3,064 (3.1%) | 0.241 |
| **Race** | | | | | | |
| White | 17,857,203 (59.9%) | 68,299 (68.4%) | **< 0.001** | 62,327 (62.5%) | 68,299 (68.4%) | **< 0.001** |
| Unknown race | 5,535,554 (18.6%) | 10,621 (10.6%) | **< 0.001** | 18,080 (18.1%) | 10,621 (10.6%) | **< 0.001** |
| Black or African American | 3,647,348 (12.2%) | 11,841 (11.9%) | **< 0.001** | 11,103 (11.1%) | 11,841 (11.9%) | **< 0.001** |
| Other Race | 1,547,991 (5.2%) | 3,751 (3.8%) | **< 0.001** | 4,348 (4.4%) | 3,751 (3.8%) | **< 0.001** |
| Asian | 1,043,983 (3.5%) | 4,344 (4.4%) | **< 0.001** | 3,362 (3.4%) | 4,344 (4.4%) | **< 0.001** |
| Native Hawaiian or other Pacific Islander | 105,139 (0.4%) | 621 (0.6%) | **< 0.001** | 341 (0.3%) | 621 (0.6%) | **< 0.001** |
| American Indian or Alaska Native | 84,035 (0.3%) | 313 (0.3%) | 0.058 | 229 (0.2%) | 313 (0.3%) | **< 0.001** |
| **Ethnicity** | | | | | | |
| Not Hispanic or Latino | 18,756,726 (62.9%) | 72,354 (72.5%) | **< 0.001** | 64,669 (64.8%) | 72,354 (72.5%) | **< 0.001** |
| Hispanic or Latino | 3,412,931 (11.4%) | 8,309 (8.3%) | **< 0.001** | 8,528 (8.5%) | 8,309 (8.3%) | 0.078 |
| Unknown Ethnicity | 7,651,596 (25.7%) | 19,127 (19.2%) | **< 0.001** | 26,593 (26.6%) | 19,127 (19.2%) | **< 0.001** |
| **Comorbidities and lifestyle factors** | | | | | | |
| Congenital malformations of the circulatory system | 135,883 (0.5%) | 1,856 (1.9%) | **< 0.001** | 410 (0.4%) | 1,856 (1.9%) | **< 0.001** |
| Diabetes mellitus | 1,140,034 (3.8%) | 17,839 (17.9%) | **< 0.001** | 5,981 (6.0%) | 17,839 (17.9%) | **< 0.001** |
| Hypotension | 205,306 (0.7%) | 9,167 (9.2%) | **< 0.001** | 978 (1.0%) | 9,167 (9.2%) | **< 0.001** |
| Hypertensive diseases | 2,478,832 (8.3%) | 42,174 (42.3%) | **< 0.001** | 13,024 (13.1%) | 42,174 (42.3%) | **< 0.001** |
| Overweight and obesity | 1,069,165 (3.6%) | 19,736 (19.8%) | **< 0.001** | 4,293 (4.3%) | 19,736 (19.8%) | **< 0.001** |
| Data are represented as mean ± SD or n (%). DS, Down syndrome. All significant values are indicated in bold, p<0.05. ^†^Demographic parameters used to match groups. | | | | | | |

| Supplemental Table 8. The incidence of cardiovascular diseases in adults without Down syndrome comparing the incidence of cardiovascular disease after treatment with anthracyclines. | | | | | | | | |
| --- | --- | --- | --- | --- | --- | --- | --- | --- |
|  | **Before matching** | | | | **After matching** | | | |
|  | **No anthracyclines, n (%)** | **Anthracyclines, n (%)** | **Odds ratio (95% CI)** | **p-value** | **No anthracyclines, n (%)** | **Anthracyclines, n (%)** | **Odds ratio (95% CI)** | **p-value** |
| Ischemic heart diseases | 1,160,167 (4.1%) | 11,988 (13.7%) | 3.75 (3.68-3.83) | **<0.001** | 6,424 (6.9%) | 11,988 (13.7%) | 2.14 (2.07-2.2o) | **<0.001** |
| Diseases of arteries, arterioles and capillaries | 947,941 (3.3%) | 11,365 (12.8%) | 4.35 (4.27-4.44) | **<0.001** | 5,113 (5.3%) | 11,365 (12.8%) | 2.61 (2.52-2.70) | **<0.001** |
| Heart failure | 730,936 (2.5%) | 10,195 (10.9%) | 4.75 (4.66-4.85) | **<0.001** | 3,876 (4.0%) | 10,195 (10.9%) | 2.92 (2.81-3.03) | **<0.001** |
| Cerebrovascular diseases | 686,193 (2.4%) | 6,810 (7.2%) | 3.22 (3.14-3.30) | **<0.001** | 3,717 (3.9%) | 6,810 (7.2%) | 1.93 (1.86-2.01) | **<0.001** |
| Diseases of veins, lymphatic vessels and lymph nodes | 791,224 (2.7%) | 20,891 (26.2%) | 12.8 (12.60-13.01) | **<0.001** | 3,519 (3.6%) | 20,891 (26.2%) | 9.47 (9.12-9.83) | **<0.001** |
| Cardiomyopathy | 313,835 (1.1%) | 6,716 (7.0%) | 7.04 (6.87-7.22) | **<0.001** | 1,597 (1.6%) | 6,716 (7.0%) | 4.59 (4.34-4.85) | **<0.001** |
| Other cardiac arrhythmias | 1,079,634 (3.7%) | 12,482 (13.9%) | 4.22 (4.14-4.30) | **<0.001** | 4,630 (4.8%) | 12,482 (13.9%) | 3.24 (3.12-3.35) | **<0.001** |
| DS, Down syndrome. All significant values are indicated in bold, p<0.05. | | | | | | | | |
